# The role of tacrolimus in women with unexplained infertility: A narrative review

**DOI:** 10.18502/ijrm.v23i5.19261

**Published:** 2025-07-29

**Authors:** Abbas Khalili

**Affiliations:** ^1^Department of Pediatrics, Shahid Sadoughi Hospital, School of Medicine, Shahid Sadoughi University of Medical Sciences, Yazd, Iran.; ^2^Hematology and Oncology Research Center, Non-Communicable Diseases Research Institute, Shahid Sadoughi University of Medical Sciences, Yazd, Iran.; ^3^Children Growth Disorder Research Center, Shahid Sadoughi University of Medical Sciences, Yazd, Iran.

**Keywords:** Infertility, Tacrolimus, Habitual abortion, Pregnancy, Assisted reproductive techniques.

## Abstract

Several autoimmune and immunological disorders can cause infertility. About 10–20% of infertility cases are related to fetal-maternal immune factors. The use of immunological treatments in women with infertility is an emerging strategy. Tacrolimus, a calcineurin inhibitor, has been shown in some studies to prevent fetal rejection and promote tolerance by inhibiting activated natural killer cells, natural killer T cells, and macrophages. In this review, we will evaluate the effects of tacrolimus on recurrent pregnancy loss and assisted reproductive technology failure. The search strategy for relevant articles was conducted in PubMed, Scopus, Google Scholar, and Web of Science databases using MeSH terms and keywords including Tacrolimus [mh], FK506, infertility [mh], abortion, spontaneous [mh], recurrent miscarriage, recurrent pregnancy loss, and recurrent implantation failure. We sought the most recent and reliable studies in the field of infertility. Tacrolimus is relatively safe and effective during pregnancy, with no major fetal-maternal complications. It is beneficial for with an elevated T helper-1/T helper-2 cell ratio. However, more studies should be designed to clarify the optimal dosage, treatment duration, and timing of initiation and cessation of tacrolimus to maximize its safety and efficacy during pregnancy.

## 1. Introduction

It is well established that various autoimmune and immunological disorders can lead to infertility. Immune cells and their regulatory mechanisms are vital for the successful implantation of allogeneic fetuses during pregnancy. While foreign fetal cells can activate cytotoxic and helper T cells, immunologic tolerance is essential for a successful pregnancy. Immune cells-including natural killer (NK) cells, dendritic cells, macrophages, and T cells, play a crucial role in embryo implantation and endometrial receptivity. Impaired immune function in the endometrium can lead to implantation failure (1–4). Certain chronic inflammations of the endometrium, such as endometriosis, can lead to infertility. The immune system is involved in the pathogenesis of endometriosis. Mucosal-associated invariant T cells play a crucial role in uterine mucosal immunity by production of immunologic cytokines such as interleukin (IL)-17, interferon gamma (INFγ), and tumor necrosis factor alpha (5, 6). Recurrent miscarriage (RM) is referred to ≥ 2 consecutive spontaneous abortions. Approximately 2.5% of couples of reproductive age experience RM. Implantation failure can occur in women undergoing assisted reproductive technology (ART). There is no consensus on the definition of recurrent implantation failure (RIF). According to the European Society of Human Reproduction and Embryology, RIF is defined as implantation failure to achieve implantation after the transfer of 3 or more euploid embryos. Approximately 10% of couples undergoing ART experience RIF (7–9). Immunologic factors contribute to successful pregnancy and ART. Studies have shown that the balance of the T helper-1/T helper-2 (Th1/Th2) cytokine ratio is a key factor in implantation and pregnancy outcomes. Some women with RIF exhibit elevated levels of Th1/Th2 cytokine ratios. Other immunologic abnormalities are also observed in these women, including altered uterine NK cells function and abnormal macrophage counts within the uterine immune environment (10, 11). Studies have shown that the immune system is very important for successful pregnancy and ART. T cells, B cells, and macrophages are immune cells that contribute to tissue homeostasis in the reproductive tract (12). About 10–20% of infertility is related to fetal-maternal immunity. Treating these women is very challenging. Although several immunosuppressive and immunomodulatory agents have been used, there are few established immunological treatments available in this clinical setting. Because of recent research in organ transplantation and cancer, our understanding of immunological factors involved in infertility treatment is increasing (13). Tacrolimus is a calcineurin inhibitor used as immunosuppressive therapy in women who have undergone solid organ transplantation (14). Tacrolimus is a macrolide antibiotic also known as FK506 (15, 16). This calcineurin inhibitor blocks calcium-dependent signaling, preventing the nuclear factor of activated T cells from migrating into the nucleus of activated T cells. Tacrolimus acts through the calcineurin-nuclear factor of activated T cells pathway in T cells, NK/NKT cells, macrophages, B cells, and dendritic cells. It is used in pregnancies complicated by systemic lupus erythematosus. Although some studies have reported that tacrolimus can cause adverse effects such as increased susceptibility to infection, elevated blood pressure, and abnormal glucose metabolism, one retrospective observational study showed that tacrolimus did not significantly affect blood pressure or glucose metabolism. However, they suggest that more data should be gathered to confirm the side effects of tacrolimus during pregnancy (17, 18). Some studies have demonstrated that tacrolimus can convert mature dendritic cells to tolerogenic dendritic cells. These findings suggest that tacrolimus may prevent fetal rejection and promote tolerance by inhibiting activated NK/NKT and macrophage cells (13). In this review, the author evaluates the effects of tacrolimus on recurrent pregnancy loss (RPL) and ART failure, drawing from the latest and most reliable studies in immunology and reproductive medicine. In this study, we conducted a comprehensive search for relevant articles using PubMed, Scopus, Google Scholar, and Web of Science databases. The MeSh headings and keywords that were used included Tacrolimus [mh], FK506, infertility [mh], abortion, spontaneous [mh], recurrent miscarriage, recurrent pregnancy loss, and recurrent implantation failure. We sought the most recent and reliable studies in the field of infertility.

## 2. Immunological aspects of tacrolimus in infertility: efficacy, complications, and safety 

Tacrolimus has recently emerged as a potential therapeutic option for women with unexplained infertility, RPL, and RIF. It appears to be effective in women with higher Th1 immune responses. Furthermore, Th1 cytokines are therapeutic targets for tacrolimus (19). Tacrolimus use has been reported in RPL, but our evidence remains insufficient. In a study of 30 pregnant women with elevated Th1/Th2 ratios treated with tacrolimus, 21 people (70%) achieved a live birth even in the first pregnancy. Women under 40 yr had a higher rate of successful pregnancy (77.8%). According to this study, tacrolimus administered at doses of 2–3 mg/day was safe during pregnancy. They concluded that tacrolimus is the treatment of choice for women with RPL (20). The safety of tacrolimus during pregnancy has been reported in women with organ transplants, with no major congenital malformations observed (21, 22). It is demonstrated that tacrolimus is safe in individuals with RIF and RPL. In a prospective study, 109 women with RIF and RPL associated with elevated Th1/Th2 ratio (CD4+ interferon gamma [IFN-γ]+/CD4+ IL-4+) were enrolled. All participants were treated with tacrolimus at doses of 1–4 mg/day before and during pregnancy. They concluded that tacrolimus treatment in women with RIF and RPL is not associated with obstetrical and perinatal complications. Of 153 women who participated in this study, 55 had RPL and 98 had RIF. Among the 98 women with RIF, 74 achieved successful pregnancies and delivered live neonates. In the RPL group, 30 women delivered live babies (23). Tacrolimus was more effective in women with RPL compared to those who did not receive treatment with tacrolimus. The researchers considered a cut-off index of 11.8 for the Th1/Th2 ratio to identify the immune imbalance. The women with RPL treated with tacrolimus had significantly lower rates of RPL and higher livebirth rates compared to those not receiving tacrolimus. However, the rate of biochemical pregnancy was significantly lower in this group. Although tacrolimus is a crucial agent for organ transplantation, it appears that in the near future, tacrolimus will become an important drug in the field of reproductive medicine (24, 25). A prospective cohort study enrolled 124 women with RIF who had Th1/Th2 ratio greater than 10.3. The women were divided into 3 groups based on their Th1 cell levels, that is, low level (Th1 < 22.8), medium level (22.8 ≤ Th1 < 28.8), and high level ( ≥ 28.8). Based on the Th1/Th2 cell ratio, these women were treated with tacrolimus at doses of 1–3 mg. The clinical pregnancy rate in the high, medium, and low groups were 33.3%, 43.9%, and 48.8%, respectively. Th1/Th2 ratio is probably an important indicator of ART prognosis in women treated with tacrolimus. There is even a case report of a successful pregnancy after 12 consecutive miscarriages (26, 27).

The beneficial effects of tacrolimus in women with polycystic ovarian syndrome (PCOS) have also been investigated. Recently, several immunologic abnormalities have been identified in women with PCOS. PCOS is commonly associated with obesity and chronic inflammation. Recent investigations have revealed abnormalities in the activation of ovarian macrophages, as well as both numerical and functional deficiencies in Th17 cells (CD4+IL-17A+) and (CD4+CD25+CD127 ${}^{\text{low}}$ ) regulatory T cells. Studies using mouse models of PCOS have shown that pre-pregnancy administration of tacrolimus can prevent reproductive complications. It has been suggested that obese and diabetic females with PCOS exhibit an imbalance in the Treg/Th17 cell ratio, a factor that may contribute to early pregnancy loss. Tacrolimus at a dose of 0.1 mg/kg was effective in promoting successful early pregnancy (28). Evidence shows that women with PCOS have chronic inflammation accompanied by elevated levels of CRP, IL-1, IL-6, tumor necrosis factor alpha, INFγ, and monocyte chemoattractant protein-1. Autoantibodies have also been reported in these women. Furthermore, evidence suggests that immunomodulation with tacrolimus may be beneficial for female infertility in murine models with PCOS. Tacrolimus can suppress systemic immune irregularity and improve endometrial receptivity (29).

Tacrolimus can significantly decrease the expression of IL-4, INFγ, and the INFγ/IL-10 ratio, while increasing the expression of leukocyte inhibitory factor, IL-10, and IL-17. Clinical pregnancy rate, implantation rate, and live birth rate in women treated with tacrolimus were 50%, 40%, and 35%, respectively. It appears that there is a positive correlation between IL-10 levels and implantation rate (30). A total of 1042 women with RIF and RM were evaluated. Of these, 485 women were assigned to the calcineurin inhibitor (tacrolimus, cyclosporine A) treatment group, and 557 were in the control group. Women in the calcineurin inhibitor group had higher live birth and clinical pregnancy rates compared to control group. Obstetrical and neonatal complications were similar in both groups. Administration of calcineurin inhibitors at low doses and for a short period did not result in serious complications (7). Normal trophoblast migration and invasion are important factors that contribute to proper placentation and successful pregnancy. Progesterone receptors play a critical role in progesterone signaling as well as trophoblast invasion and migration. Abnormal progesterone receptor expressions may lead to placentation failure. Low doses of tacrolimus could normalize progesterone receptor expression and improve placentation and complications in women with immune-mediated infertility. However, the exact mechanism underlying this action remains unclear. The mode of action of tacrolimus on the extravillous trophoblast cell line has been identified, indicating that a low dose of tacrolimus (10 ng/ml) can significantly stimulate extravillous trophoblast cells (HTR-8/SVneo cells). It has been suggested that a low dose of tacrolimus can positively influence immune-mediated pregnancy complications through immune-independent mechanisms in the first trimester (31). 149 women with refractory recurrent spontaneous abortion and elevated Th1/Th2 ratio or IL-33/ST2 levels in peripheral blood were evaluated. They were randomly divided into 2 groups: tacrolimus (at a dose of 0.05 ∼ 0.1 mg/kg/day) and a placebo group (74 women). The rate of normal live delivery was 80% in the tacrolimus group and 63% in the placebo group. The Th1/Th2 ratio and IL-33/ST2 levels were significantly lower in the tacrolimus group (p < 0.05). The results were consistent with the hypothesis that tacrolimus treatment may be an effective approach for refractory recurrent abortion (32). According to several studies, tacrolimus is considered relatively safe during pregnancy and lactation. Because the pharmacokinetics of tacrolimus change during pregnancy, higher doses may be required to maintain therapeutic blood levels. Therefore, serum level monitoring is essential for appropriate dose adjustment (33–35). A systematic review and meta-analysis evaluated 98 studies and analyzed 4450 pregnant women treated with calcineurin inhibitors (tacrolimus, cyclosporine) for various indications. The live birth rate was 82.1%, while the rate of spontaneous abortion was 11.7% (36). In one cohort, organ-specific autoimmunity was correlated with NK cells. However, it was suggested that further investigations are necessary to clarify the role of NK cells in autoimmune disorders (37). Among 249 individuals with 2 or more implantation failures and peripheral NK cell levels exceeding 12%, pregnancy outcomes were assessed in 60 women who received a combination of tacrolimus and low molecular weight heparin (LMWH) compared to 85 women treated with LMWH alone. The remaining women comprised the control group and did not receive any medication. A combination of tacrolimus and LMWH has been shown to improve the live birth rates and clinical pregnancy rates as well as reduce spontaneous miscarriage in women with elevated peripheral NK cell levels (38). Some cases of unexplained pregnancy loss and preeclampsia are associated with maternal-fetal immunologic abnormalities. Tacrolimus may help prevent unexplained pregnancy loss, preeclampsia, and stillbirth by modulating the Th1/Th2 ratio (39).

## 3. Conclusion

According to this review, tacrolimus appears to be relatively safe and effective during pregnancy, with no major maternal or fetal complications reported. Most studies suggest that tacrolimus is beneficial for women with an elevated Th1/Th2 cell ratio. Due to limited number of studies in this area, further research is needed to confirm the efficacy and safety of tacrolimus in treating spontaneous abortion, RIF, and RM related to immunologic dysregulation. Future studies should be designed to determine the optimal dosage, treatment duration, timing of initiation, and appropriate point of discontinuation for tacrolimus therapy.

##  Data Availability

Data supporting the findings of this study are available upon reasonable request from the corresponding author.

##  Conflict of Interest

The author declares no conflict of interest.
